# *Escherichia coli* Causing Recurrent Urinary Tract Infections: Comparison to Non-Recurrent Isolates and Genomic Adaptation in Recurrent Infections

**DOI:** 10.3390/microorganisms9071416

**Published:** 2021-06-30

**Authors:** Karen Leth Nielsen, Marc Stegger, Kristoffer Kiil, Berit Lilje, Karen Ejrnæs, Rikke Fleron Leihof, Line Skjøt-Rasmussen, Paul Godfrey, Tor Monsen, Sven Ferry, Anette M. Hammerum, Niels Frimodt-Møller

**Affiliations:** 1 Department of Clinical Microbiology, Rigshospitalet, 2100 Copenhagen, Denmark; karen.leth.nielsen.01@regionh.dk; 2 Department of Bacteria, Parasites and Fungi, Statens Serum Institut, 2300 Copenhagen, Denmark; MTG@ssi.dk (M.S.); krki@ssi.dk (K.K.); beli@ssi.dk (B.L.); karen.ejrnaes.03@regionh.dk (K.E.); rfel@novonordisk.com (R.F.L.); liskmail@icloud.com (L.S.-R.); ama@ssi.dk (A.M.H.); 3 Department of Pathology, Herlev Hospital, 2730 Herlev, Denmark; 4 Analytical Development, Novo Nordisk, 2880 Måløv, Denmark; 5 Animal Health Innovation, Chr. Hansen, 2970 Hørsholm, Denmark; 6 Genome Sequencing and Analysis Program, Institute of Technology, Broad Institute of Harvard and Massachusetts, Cambridge, MA 02142, USA; pagodfrey@gmail.com; 7 Department of Clinical Microbiology, University of Umeå, 901 04 Umeå, Sweden; tor.monsen@umu.se (T.M.); sven.ferry@umu.se (S.F.)

**Keywords:** whole-genome sequencing, urinary tract infection, mobilome, single nucleotide polymorphisms, adaptation, genomics, *Escherichia coli*

## Abstract

Recurrent urinary tract infection (rUTI) remains a major problem for many women and therefore the pursuit for genomic and phenotypic traits which could define rUTI has been ongoing. The present study applied a genomic approach to investigate recurrent urinary tract infections by comparative analyses of recurrent and non-recurrent *Escherichia coli* isolates from general practice. From whole-genome sequencing data, phylogenetic clustering and genomic traits were studied on a collection of isolates which caused recurrent infection compared to non-recurrent isolates. In addition, genomic variation between the 1st and following infection was studied on a subset of the isolates. Evidence of limited adaptation between the recurrent infections based on single nucleotide polymorphism analyses with a range of 0–13 non-synonymous single nucleotide polymorphisms (SNPs) between the paired isolates. This included an overrepresentation of SNPs in metabolism genes. We identified several genes which were more common in rUTI isolates, including nine fimbrial genes, however, not significantly after false-discovery rate. Finally, the results show that recurrent isolates of the present dataset are not distinctive by variation in the core genome, and thus, did not cluster distinct from non-rUTI isolates in a SNP phylogeny.

## 1. Introduction

Recurrent urinary tract infections (rUTIs) occur in up to 25–40% of all UTI episodes and is often caused by the same strain as the index strain, most often uropathogenic *Escherichia coli* (UPEC) [1,2]. rUTIs are generally defined as ≥2 episodes of UTI within six months or three episodes in 12 months [3]. The recurrent infections proposedly take place by two major mechanisms: (i) Reintroduction of the same isolate, e.g., by contamination from vagina or rectum, or (ii) persistence where antibiotics do not clear infection possibly due to internal bladder colonies (IBCs), of which reintroduction is the most common [4,5]. Several host factors have been correlated to UTI and rUTIs, which include increased susceptibility for vaginal colonization with uropathogens, increased adherence of UPEC to uroepithelial cells, post-menopause and sexual intercourse [5,6].

Several studies have investigated the virulence of recurrent *E. coli* without identification of any traits associated to recurrent uropathogenic *E. coli* [4,7,8,9,10]. It has been shown that specific virulence factors and clonal groups of *E. coli* were over-represented in either rUTI or cure based on PCR studies of specific genetic traits [11]. Recurrence has been associated with specific antibiotic resistance traits and sequence types [7,9] or significantly related to genes associated with biofilm production [12]. Today, whole-genome sequencing (WGS) enables an unbiased search for differences in genetic traits and includes not only known virulence traits but rather a whole-genome scale, i.e., gene content, single nucleotide polymorphisms (SNPs) and k-mers. rUTIs with the same strain causing both the initial and the recurrent infection have only been sparsely analyzed on a genomic level with respect to genomic changes between the two infections as well as comparison of rUTI to non-rUTI. Chen et al. [13] investigated adaptation between rUTI in one patient and identified few SNPs between the isolates. Thänert et al. [4] recently published a study on four rUTI patients, where the infections were caused by antimicrobial-resistant *E. coli* isolates and found little diversity between infections and did not identify any specific virulence traits in rUTI isolates when compared to non-recurrent UTI isolates. Thus, the aspect of genetic traits and rUTI has so far not been studied on any larger collection of isolates, nor on collections of antimicrobial susceptible isolates. Comparing recurrent episodes of UTI on a larger set of isolates will additionally clarify whether the isolates develop into specialized UTI isolates, or whether the isolate is fit for colonization without distinct adaptation to the new environment. A previous study regarding adaptation between fecal carriage and UTI infection showed very limited adaptation between the two environments, indicating that the *E. coli* is well-adapted to both environments prior to causing UTI [14].

Here, we performed genomic analyses on a larger collection of recurrent and non-recurrent *E. coli* from well-characterized lower urinary tract infections, with the aim of (i) comparing recurrent and non-recurrent isolates in terms of relatedness and genetic content, and (ii) identify genomic plasticity and adaptation by comparing *E. coli* isolates between the first and recurrent infections in individual women.

## 2. Materials and Methods

### 2.1. Study Population

The present study is based on a strain collection from a multicenter, randomized, double-blind, placebo-controlled comparative study of different dosing regimens of pivmecillinam (piv-amdinocillin) for lower UTI [15]. Criteria for inclusion were women over 18 years of age with symptoms of lower UTI, i.e., urgency, dysuria, suprapubic pain or loin pain. Exclusion criteria encompassed antibiotic therapy for UTI within the last month, participation in other studies within last three months, known/suspected penicillin allergy, genital infection, complicating factors (diabetes or abnormality of the urinary tract), one or more signs of pyelonephritis (pyrexia: ≥38.5 °C, CRP ≥25 mg/L, kidney tenderness by palpation), urine incontinence requiring catheter/pads, pregnancy/planned pregnancy or previous participation in the study. Patients were randomized to pivmecillinam at 200 mg three times a day (TID) for 7 days, 200 mg twice a day (BID) for 7 days, 400 mg BID for 3 days or placebo. Patients were evaluated clinically and bacteriologically with a mid-stream urine sample at day 1 (inclusion), days 8–10 (first follow-up) and days 35–49 (second follow-up). In the parent study, the majority (86%) of the cases with significant bacteriuria (≥10^3^ colony forming units (CFU)/mL) at follow-up among women with *E. coli* at inclusion, also had *E. coli* bacteriuria at follow-up. In a previous study [16], a subgroup (156 patients) was chosen according to the following criteria: from the pivmecillinam treatment group all patients showing significant *E. coli* bacteriuria and symptoms of UTI at inclusion and at one or both follow-up visits were selected. Recurrent isolates of this study were included from the mecillinam-treatment groups (i.e., two infections within 49 days following relevant treatment) as positive for the same *E. coli* at initial sampling as well as at first or second follow-up, as determined by pulsed-field gel electrophoresis (PFGE) [16]. Cure was defined as negative urine sample at follow-up or positive for a different *E. coli* or other bacteria than the initial infection.

### 2.2. Bacterial Isolates

For the present study we randomly selected from the 156 previously PFGE-typed *E. coli* strain population same-strain recurrent isolates from the first patient-visit (*n* = 45) and isolates from patients who did not suffer recurrence after antibiotic treatment (*n* = 43) (Supplementary Table S1 for overview over isolates). For analysis of within-patient adaptation related to rUTI, we additionally selected pairs of matching recurrent isolates from the 1st and following infection (*n* = 35 pairs). These were selected randomly within the collection of same-strain recurrent isolates to investigate adaptation between first and second sampling. Thus, the *E. coli* population analyzed in this study did not represent any patients in the placebo group of the original study.

### 2.3. Sequencing, Assembly and Annotation

The isolates in this study were subject to whole-genome sequencing on HiSeq (Illumina, 2 × 100 base-pair (bp)) using 180-bp fragment libraries and 3-kb mate-pair libraries for scaffolding [14,17]. The isolates from the second infections were sequenced using paired-end libraries (*n* = 35) on a Miseq (2 × 250 bp), and a subset of these were sequenced with both paired-end and mate-pair libraries (*n* = 20) to further advance investigations into the mobilome. Mate-pair genomes were assembled using ALLPATHS-LG and annotated as previously described [17]. Isolates sequenced with paired-end only were assembled using VelvetOptimiser [18]. Genome quality and sequencing methods are listed in Supplementary Table S1.

### 2.4. Phylogeny and Pairwise SNPs

A phylogeny was constructed using Parsnp [19] for all 1st isolates causing rUTI (*n* = 45) and isolates from patients with non-recurrent infections (*n* = 43) [19]. The tree was annotated with metadata using CLCbio’s Genomics Workbench v8. Pairwise SNP distances were identified using Biomatters Geneious v9.1.7 using the genome from the initial infection as reference for the read mapping of the secondary infection isolate [14]. Analysis parameters were minimum coverage of 20-fold and with a minimum variant frequency of 0.9. SNPs were manually inspected by visualization of the reference mapping using Geneious as previously described [14].

### 2.5. Accessory Genome Content and Typing

Prokka v1.2 [20] was used to annotate genes with default settings followed by analysis using Roary v3.6.0 [21] to determine the pan-genome across the collection and hence to define gene “presence/absence” in individual isolates. In Roary, all options were default except paralogs, that were set not to split. Alignment of the core genes were obtained in Roary using PRANK and MAFFT. Only genes present in <95% and >5% of the samples were kept for further analyses, this reduced the number of genes from 16,880 to 4062. These genes were investigated for overrepresentation to either group, using a Fisher’s exact test in R v3.6.2. *p*-values were corrected for multiple testing with the false discovery rate (FDR) method. To investigate if a combination of genes could predict UTI recurrence we performed Discriminant Analysis of Principal Components (DAPC) [22] from the R package adegenet v. 2.1.3. The optimal number of principal components (PCs) was found using the function xvalDAPC which performs multiple cross validations for DAPC analysis. Number of replications were set to 1000, for PCs: 5, 10, 15, 20, 25 and 30, otherwise default settings were used.

For a k-mer analysis, all assembled genomes were broken down to 30-mers and added to a dictionary using python v2.7.10. Each unique k-mer was investigated for overrepresentation to either group, using Fisher’s exact test in R v3.3.1 with corrected *p*-values for multiple testing using FDR. If the k-mer, or reverse complement of the k-mer already was present in the dictionary, the occurrence was incremented by one. Each k-mer was only counted once per sample to avoid that few samples with long k-mer repeats would skew the results. The k-mers did not span contig junctions. To type the isolates MLST v2.0 was applied and *in silico* phylogrouping according to Clermont et al., 2013 [23]. Twenty paired genomes were also compared using whole-genome alignments (WGA) using progressive Mauve implemented in Geneious v9.1.7 using default settings. Mauve regions were verified by reference mapping and coverage inspected (minimum 10× coverage). 

### 2.6. Ethical Approval

The original study [15] was conducted in accordance with the Swedish Medical Product Agency guidelines and was approved by the Agency 1995 03 01 (Dnr 151: 01783/94) as well as by the Ethics Committee of Umea University 1995 03 07 (Dnr 93–178).

## 3. Results and Discussion

Our aim with the present study was to increase our understanding of the genetics behind rUTI by performing genomic analyses on a larger collection of recurrent and non-recurrent isolates from well-characterized lower urinary tract infections. This with the aim of (i) comparing recurrent and non-recurrent isolates in terms of relatedness and genetic content, and (ii) identify genomic plasticity and adaptation by comparing isolates between the first and recurrent infections in individual women. If rUTI isolates can be distinguished genetically, this could potentially improve diagnostics and treatment of these infections.

### 3.1. Adaptation between First and Second UTI

**Figure 1:**
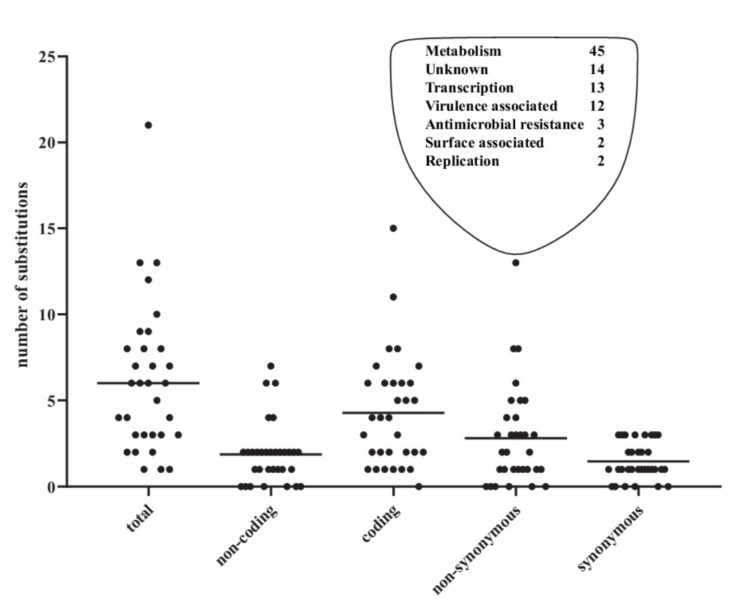
Number of SNPs between the first and recurrent infection in 32 matching UTI pairs and the number of non-synonymous SNPs divided into categories based on annotations. Lines illustrate medians.

Of the selected PFGE matching pairs of isolates from individual patients, 32 of the 35 (91%) were similar based on WGS with a median of six SNPs/pair (range 1–21) and a median of two non-synonymous SNPs (NSY) per pair (range 0–13) (Figure 1).

**Figure 2:**
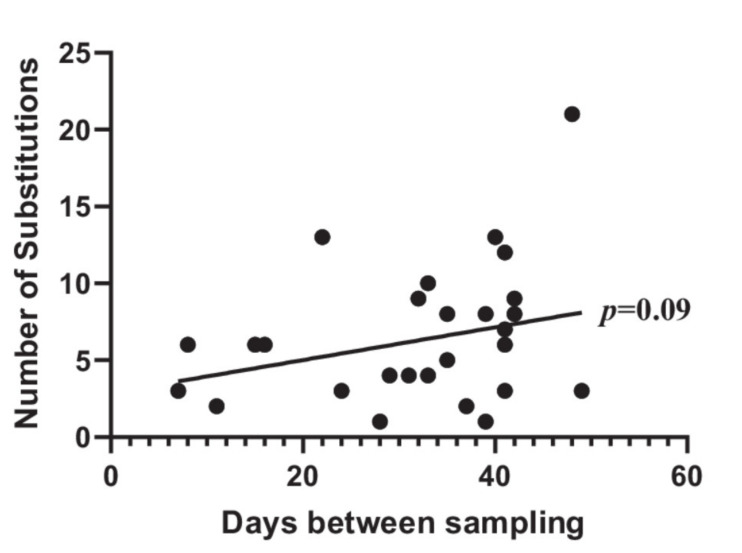
Number of SNPs in correlation to time between urine samples of the same patient. Line is linear regression line, tested for slope significance.

The remaining three non-identical pairs were discarded from further analyses due to 36,108, 97,781 and 100,224 SNPs, respectively, between the first and following episode, distributed across the whole genome. We investigated whether the number of SNPs was correlated to the number of days between the paired samples (Figure 2).

There was a slight trend, however, not significant (*p* = 0.09) towards more SNPs with the longer time between the samples (Figure 2). In total, 91 NSY SNPs were identified in the 32 pairs. Of these, 45 were positioned in metabolism-associated genes (Figure 1, Table S2). The number of SNPs in metabolism genes is striking as they constitute 49.5% (45/91 NSY SNPs) of all NSY SNPs across the pairs of isolates from recurrent infections. Overall, across the collection, 42 different metabolism genes had NSY SNPs (highlighted in bold, Table S2) compared to 45 genes with other functions among an average of 4806 genes per isolate. With metabolism genes constituting approximately 30% of the coding *E. coli* genome [24], this yields 1441 (30% of 4806 genes) metabolism associated genes per isolate. Hence, an overrepresentation of SNPs was associated to these genes compared to the total number of genes (Fisher’s exact test, *p* = 0.0004). That metabolism genes are subject to mutation could be correlated to a change in nutrients available. To highlight a few, one gene, encoding the protein NarX, caught NSY SNPs in a total of three pairs. The NSY were not in identical positions, but these data strongly indicate that this gene is subject to adaptation during UTI. The protein NarX is part of a two-component regulatory system adapting to amount of nitrate in the environment [25]. Nitrate is present in urine and any adaptation to adjust uptake of nitrate available for the bacteria could indicate change in aerobic/anaerobic metabolism. Another protein, Slp, which is activated upon carbon starvation had a NSY SNP in one isolate. Combined, the metabolism genes with NSY SNPs indicate that changes in metabolic components are important in order to adapt to the urinary environment. However, we only observe a few SNPs in each pair, so this could be considered a genetic fine-tuning, in order to increase fitness and survival chances in the bladder environment with new nutrients available, compared to the likely preceding intestinal and/or vaginal environment.

We investigated gene loss on all isolates from whole-genome alignments using progressive Mauve with subsequent verification using reference read mapping. Here 18 of the 20 (90%) mate-pair isolate pairs had identical gene content in their accessory genome when comparing isolates of the first and following infection and only two isolates (#3889 and #4076) had changes in gene content. Isolate #3889 gained 59,769 bp of plasmid material (top BLAST hit: KT754167, 99.1% sequence identity, 75.5% sequence coverage) as well as a 6253 bp plasmid region (top BLAST hit KM085450, 97.7% sequence identity, 55% sequence coverage). Whether these two acquisitions are linked cannot be established with certainty based on the current genome assembly. The gained material included prophage-related material so a phage acquisition cannot be excluded. Isolate #4076 had an additional copy of a urea transporter in the isolate from the recurrent episode (similar to protein ID: VED05424.1 of GenBank ID: LR134237).

Noteworthy, the dataset described in this study revealed only minute differences between the first and second infection in individual patients indicating limited adaptation and the isolates with different gene content between infections were isolates sampled the most far apart in time when considering the complete isolate collection (48 and 39 days apart, respectively). One explanation for the lack of genetic changes in the majority of the isolates could be that the period of time between the sampling was not long enough to study this adaptation for many of the isolates. A recent study on three antibiotic-resistant *E. coli* from rUTI identified <10 SNPs between the first and second infection [4]. Our data support this limited variation but also expands upon this by showing that this is also the case in a larger susceptible population.

### 3.2. Do rUTI Isolates Differ Genetically from Non-rUTI Isolates?

**Figure 3:**
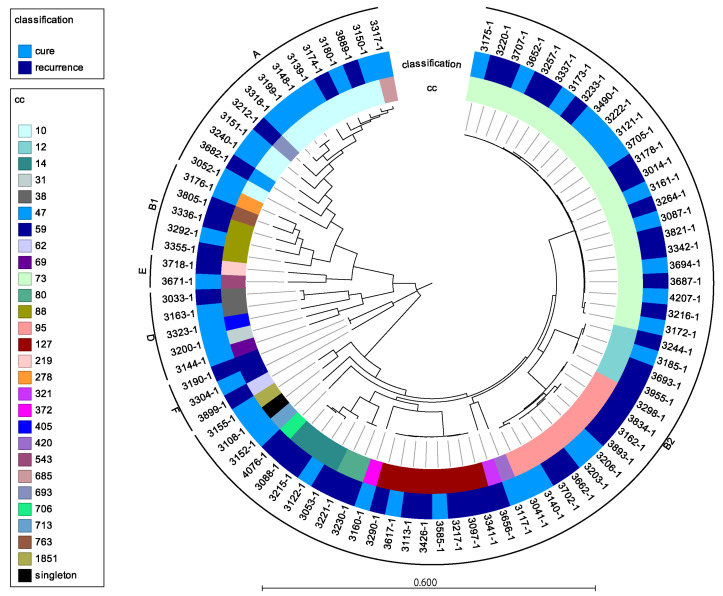
Midpoint-rooted phylogenetic clustering of recurrent and non-recurrent *E. coli*. Phylogenetic groups are illustrated along the periphery of the tree. Scale bar: substitutions/site. CC: clonal complexes based on MLST typing.

The SNP-based analysis across the data set of rUTI isolates and non-rUTI revealed that the isolates clustered in the phylogeny based on sequence type and phylogroup with many isolates belonging to common urinary and fecal sequence types (illustrated as clonal complexes, CC’s), with ST73 as the most common type (Figure 1). In addition, isolates causing recurrent infections did not cluster distinct from isolates causing single UTIs (Figure 3). The data illustrated that isolate causing rUTI did not constitute distinct monophyletic clusters, and hence, did not evolve independently compared to non-recurrent isolates.

To analyze the accessory genome, we compared non-core genes across both rUTI and non-rUTI isolates with Roary (*n* = 4062), identifying any differences in presence/absence of genes in the rUTI and non-rUTI group. In addition to this we analyzed all 30 bp k-mers (*n* = 29,746,686) in the assembled genomes to identify overrepresentation in one of the groups.

Both analyses revealed no genes or k-mers significantly overrepresented in the recurrence group compared to the non-recurrence group after adjusting for multiple testing. Unbiased search for genes and k-mers throughout the genomes, indicated that no unique traits distinctively classify *E. coli* as recurrent and non-recurrent. This could reflect the current dataset, and/or caused by lack of statistical power from the large collection of genes in the included diverse study population. Multivariate analysis (DAPC) was performed to investigate if a combination of multiple genes could predict rUTI. Multiple cross-validations revealed that the optimal number of PCs was 15 PCs, which on average gave a mean success rate of 0.52. A random model would predict correctly in ca. 50% of cases, which means that a combination of genes could not predict outcome in this dataset and further DAPC analysis was terminated. The isolates in this study were all from the same cohort, however, they spanned 6 of the 8 known phylogroups. This diversity relates to the large pangenome and thus the number of multiple tests to be performed, with lowered power as a result.

The Roary analyses identified genes which were overrepresented in the rUTI group compared to the non-rUTI group, although not significantly after adjusting the multiple testing by FDR (Table 1). The results indicate, based on our data, that prediction of recurrence based on any *E. coli* genomic traits alone is not readily achieved. 43/50 genes with lowest *p*-values pre-FDR were linked to genes overrepresented in the rUTI group (Table 1). Interestingly, these genes represented fimbriae (including seven genes from the S/F1C fimbrial cluster), lipopolysaccharide (LPS), biosynthesis, toxin/antitoxin systems, type VI secretion system, metabolism, as well as a secretion pathway (Table 1). Many of the genes identified are linked to the same clusters within each category, including LPS synthesis, metabolism and the S/F1C fimbrial cluster (Table 1). Fimbrial genes, type VI secretion, toxin/antitoxin systems have been linked to virulence of *E. coli* in previous studies [26,27,28]. The S/F1C fimbria has been associated with biofilm production [29], a feature which has been correlated to internal bladder colonization and rUTI previously [30]. This fimbria is not essential in order to bind to the epithelial lining of the bladder, however, likely contributes to virulence of the isolates and could influence on persistence of the infection. This should be investigated in a larger study. In a previous study we have identified genes of the type VI secretion system to be overrepresented in UTI isolates compared to fecal isolates [14], which supports the present data.

Seven of the genes were more common in the non-rUTI group. These were genes responsible for LPS biosynthesis including genes from the *waa*-cluster (*waaO*, *waaY*, *waaZ*, *waaJ* and *htrL*), *pipB* (type III secretion system protein) and a gene encoding a phage protein (Table 1).

In this study, we analyzed genetic variation and content for a collection of isolates which caused UTI and rUTI. The present study compares a large collection of whole-genome sequenced *E. coli* isolates for in-depth analyses of genetic content in rUTI and non-rUTI isolates. A recent study on a smaller set of antimicrobial resistant isolates described rUTI isolates as similar to non-recurrent isolates with respect to virulence factors and phylogenetic distribution based on three *E. coli* rUTI pairs [4]. The present study elaborates on this study by describing genetic comparison in a large collection of both rUTI and non-rUTI isolates, enabling higher resolution during the analyses compared to the previous studies. Previously, we identified that healthy controls with no previously reported UTI carried genetically similar isolates in the intestinal microbiota compared to patients with a concurrent UTI [31]. The current study is in line with these results, as the findings of this study also indicate large genetic overlaps between isolates of UTI and rUTI.

Whether the phenotypes differ between recurrent and non-recurrent isolates is beyond the scope of this study, but is a relevant follow-up study. The lack of major genetic differences between the rUTI and cure group indicates the complex interplay between bacterial infection capabilities, treatment success and host factors are likely to determine recurrence rather than specific genetic characteristics of the *E. coli* bacteria itself. A larger study on hundreds or thousands of isolates might enable the power of an unbiased search through the accessory genome to identify significant traits of recurrence. Oppositely, the pangenome is likely to increase with increasing number of isolates, decreasing the likelihood of identifying significant traits for rUTI. A study on specific lineages (e.g., ST73 or ST131) on a larger collection of isolates could be another option in identifying significant genes for rUTI, as this would limit the pangenome. Future studies combining the host-genomics or transcriptomics with the bacterial genomics and microbiome studies of both urinary, vaginal and fecal environment will perhaps enable classification of pathotypes and predict recurrence.

### 3.3. Limitations

Limitations of this study include that we only sequenced one isolate per woman per incident. It is possible that the observed variation between the isolate pairs was already present at the first time of sampling. However, we have previously described intraclonal variation in both fecal and urinary environment to be sparse [32], and the transition from the fecal environment to the urinary tract has also been shown to include only minor adaptation [14]. The isolates were collected from a limited area of Sweden, which could be a possible limitation, however, the phylogenetic diversity of the isolates in this study is large and represents a broad range of clones from six different phylotypes.

## 4. Conclusions

Our findings indicate sparse genomic adaptation in some consecutive rUTIs by exchange of mobile elements and via SNPs primarily found in metabolism-associated genes. Our detailed comparison of rUTI and non-recurrent isolates, however, did not identify any significantly associated genetic factors or exhibited any distinct phylogenetic clustering.

## Figures and Tables

**Table 1:** Comparison of accessory genome of rUTI and non-rUTI isolates. Listed are top 50 *p*-values below 0.01 before false-discovery rate (FDR).

Gene Annotation	Function	*p*-Value	*p*-Value FDR	Cure *n* (%)	rUTI *n* (%)	Isolate_Locus Tag
**Overrepresented in rUTI isolates**						
Fimbria-related genes						
*papB*	S/F1C fimbrial transcriptional regulator	0.0062	0.619	14 (33)	28 (62)	3244-2_K_03164
*sfaH*	S-fimbrial protein subunit	0.0072	0.619	2 (5)	12 (27)	3244-2_K_03157
*mprA*	Transcriptional repressor	0.0072	0.619	26 (60)	39 (87)	3244-2_K_03155
*sfaF*	S/F1C fimbrial biogenesis usher protein	0.0118	0.619	15 (35)	28(62)	3244-2_K_03160
*sfaE*	S/F1C fimbrial biogenesis chaperone	0.0118	0.619	15 (35)	28 (62)	3244-2_K_03161
*sfaG*	S/F1C fimbrial adhesin minor pilin SfaG/FocF	0.0118	0.619	15 (35)	28 (62)	3244-2_K_03159
*sfaD*	S/F1C fimbrial minor subunit	0.0118	0.619	15 (35)	28 (62)	3244-2_K_03162
*ydiV*	Putative anti-FlhC(2)FlhD(4) factor YdiV	0.0118	0.619	15 (35)	28 (62)	3244-2_K_03156
*hifA*	fimbrial protein	0.0095	0.619	11 (26)	24 (53)	3244-2_K_02738
Other virulence-related genes						
*shlB*	Hemolysin transport	0.0100	0.619	16 (37)	30 (67)	3230-3_K_03314
*cbeA*	Toxin/antitoxin system	0.0128	0.619	33 (77)	43 (96)	3244-2_K_03419
*yeeW*	Toxin/antitoxin protein	0.0106	0.619	28 (65)	40 (89)	3244-2_K_03421
*yeeJ*	Adhesin	0.0079	0.619	29 (67)	41 (91)	3244-2_K_00508
*wgrG*	Type VI secretion system protein	0.0088	0.619	4 (9)	15 (33)	3244-2_K_04080
*klcA*	Anti-restriction protein	0.0068	0.619	34 (79)	44 (98)	3014-3_K_04082
*cbtA*	Toxin/antitoxin system	0.0064	0.619	32 (74)	43 (96)	3244-2_K_04704
LPS-biosynthesis						
*gspA_3*	Secretion pathway protein	0.0015	0.619	30 (70)	43 (96)	3244-2_K_02772
*gspA_4*	Secretion pathway protein	0.0015	0.619	30 (70)	43 (96)	3244-2_K_00614
*rfaY*	Lps biosynthesis protein	0.0015	0.619	30 (70)	43 (96)	3244-2_K_02773
*epsJ*	Glycosyltransferase	0.0028	0.619	26 (60)	40 (89)	3244-2_K_02775
*gspA_2*	Secretion pathway protein	0.0028	0.619	26 (60)	40 (89)	3244-2_K_02774
*rfaL*	Lps biosynthesis protein	0.0028	0.619	26 (60)	40 (89)	3244-2_K_02776
Phage-related genes						
*-*	Bacteriophage Replication protein O	0.0056	0.619	27 (63)	40 (89)	3014-3_K_04219
*-*	Phage replication protein	0.0057	0.619	23 (53)	37 (82)	3014-3_K_04220
*alpA*	Prophage regulatory protein	0.0007	0.619	20 (47)	37 (82)	3244-2_K_03100
Metabolism-related genes						
*bglA*	Metabolism protein	0.0079	0.619	29 (67)	41 (91)	3244-2_K_00072
*bglH*	Metabolism protein	0.0079	0.619	29 (67)	41 (91)	3244-2_K_00073
*yidP*	Transcriptional regulator	0.0079	0.619	29 (67)	41 (91)	3244-2_K_00077
*yfjR*	Transcriptional regulator	0.0082	0.619	20 (46)	34 (76)	3244-2_K_03712
*ykfF*	Hypothetical protein	0.0089	0.619	19 (44)	33 (73)	3244-2_K_03710
	Hypothetical protein	0.0017	0.619	20 (47)	36 (80)	3244-2_K_03711
*rspA*	Starvation sensing protein	0.0038	0.619	25 (58)	39 (87)	3244-2_K_01532
*yjjL*	Inner membrane transport protein	0.0038	0.619	25 (58)	39 (87)	3244-2_K_01531
*-*	Putative inner membrane protein	0.0024	0.619	23 (53)	38 (84)	3244-2_K_03387
*-*	Cytoplasmic protein	0.0047	0.619	24 (56)	38 (84)	3244-2_K_03386
*rbsK*	Ribokinase	0.0047	0.619	24 (56)	38 (84)	3244-2_K_03385
Replication						
*crfC*	Replication protein	0.0122	0.619	23 (53)	36 (80)	3244-2_K_03407
	Chemotaxis protein	0.0057	0.619	23 (53)	37 (82)	3244-2_K_03408
Other and unknown function						
*tnpB*	Transposase	0.0098	0.619	26 (60)	14 (31)	3805-3_K_04559
						
*ompD*	Porin protein	0.0057	0.619	23 (53)	37 (82)	3244_2_K_00430
*-*	Hypothetical protein	0.0118	0.619	15 (35)	28 (62)	3244-2_K_03094
*-*	Hypothetical protein	0.0137	0.619	22 (51)	35 (78)	3244-2_K_03400
*higA*	Toxin/antitoxin system	0.0122	0.619	23 (53)	36 (80)	3244-2_K_00724
**Overrepresented in non-rUTI isolates**						
*pipB*	Type III secretion system protein	0.0106	0.619	15 (35)	5 (11)	3212-3_K_02120
*-*	Hypothetical protein (prophage)	0.0112	0.619	6 (14)	0 (0)	MN187550_Orf34
*waaZ*	Lps biosynthesis	0.0112	0.619	6 (14)	0 (0)	CP046003_GJD94_01225
*waaY*	Lps biosynthesis	0.0112	0.619	6 (14)	0 (0)	CP046003_GJD94_01220
*waaJ*	Lps biosynthesis	0.0112	0.619	6 (14)	0 (0)	CP046003_GJD94_01215
*waaO*	Lps biosynthesis	0.0112	0.619	6 (14)	0 (0)	CP046003_GJD94_01210
*yibB*	RfaH-regulated high-temperature protein	0.0032	0.619	10	1	CP046003_GJD94_01255

## Data Availability

The data presented in this study are openly available in Genbank/ENA under the accession numbers listed in Supplementary Table S1.

## Supplementary Materials

The following are available online at https://www.mdpi.com/article/10.3390/microorganisms9071416/s1, Table S1: Overview of isolates in the study: Infection type, phylogroup, MLST clonal complex (CC), sequencing method, average sequencing depth, number of scaffolds and Genbank/ENA ID, Table S2: Coding SNPs identified between pairs.

## References

[1] Mabeck C.E. (1972). Treatment of uncomplicated urinary tract infection in non-pregnant women. Postgrad. Med. J..

[2] Russo T.A., Stapleton A., Wenderoth S., Hooton T.M., Stamm W.E. (1995). Chromosomal restriction fragment length polymorphism analysis of *Escherichia coli* strains causing recurrent urinary tract infections in young women. J. Infect. Dis..

[3] Hooton T.M. (2001). Recurrent urinary tract infection in women. Int. J. Antimicrob. Agents.

[4] Thänert R., Reske K.A., Hink T., Wallace M.A., Wang B., Schwartz D.J., Seiler S., Cass C., Burnham C.-A.D., Dubberke E.R. (2019). Comparative Genomics of Antibiotic-Resistant Uropathogens Implicates Three Routes for Recurrence of Urinary Tract Infections. MBio.

[5] Forde B.M., Roberts L.W., Phan M.-D., Peters K.M., Fleming B.A., Russell C.W., Lenherr S.M., Myers J.B., Barker A.P., Fisher M.A. (2019). Population dynamics of an *Escherichia coli* ST131 lineage during recurrent urinary tract infection. Nat. Commun..

[6] Foxman B., Frerichs R.R. (1985). Epidemiology of urinary tract infection: II. Diet, clothing, and urination habits. Am. J. Public Health.

[7] Karami N., Lindblom A., Yazdanshenas S., Lindén V., Åhrén C. (2020). Recurrence of urinary tract infections with extended-spectrum β-lactamase-producing *Escherichia coli* caused by homologous strains among which clone ST131-O25b is dominant. J. Glob. Antimicrob. Resist..

[8] Kärkkäinen U.M., Ikäheimo R., Katila M.L., Siitonen A. (2000). Recurrence of urinary tract infections in adult patients with community-acquired pyelonephritis caused by *E. coli*: A 1-year follow-up. Scand. J. Infect. Dis..

[9] Li D., Reid C.J., Kudinha T., Jarocki V.M., Djordjevic S.P. (2020). Genomic analysis of trimethoprim-resistant extraintestinal pathogenic *Escherichia coli* and recurrent urinary tract infections. Microb. Genom..

[10] Soto S.M., Smithson A., Martinez J.A., Horcajada J.P., Mensa J., Vila J. (2007). Biofilm formation in uropathogenic *Escherichia coli* strains: Relationship with prostatitis, urovirulence factors and antimicrobial resistance. J. Urol..

[11] Johnson J.R., O’Bryan T.T., Delavari P., Kuskowski M., Stapleton A., Carlino U., Russo T. (2001). A Clonal relationships and extended virulence genotypes among *Escherichia coli* isolates from women with a first or recurrent episode of cystitis. J. Infect. Dis..

[12] Soto S.M., Smithson A., Horcajada J.P., Martinez J.A., Mensa J.P., Vila J. (2006). Implication of biofilm formation in the persistence of urinary tract infection caused by uropathogenic *Escherichia coli*. Clin. Microbiol. Infect..

[13] Chen S.L., Wu M., Henderson J.P., Hooton T.M., Hibbing M.E., Hultgren S.J., Gordon J.I. (2013). Genomic Diversity and Fitness of *E. coli* Strains Recovered from the Intestinal and Urinary Tracts of Women with Recurrent Urinary Tract Infection. Sci. Transl. Med..

[14] Nielsen K.L., Stegger M., Godfrey P.A., Feldgarden M., Andersen P.S., Frimodt-Møller N. (2016). Adaptation of *Escherichia coli* traversing from the faecal environment to the urinary tract. Int. J. Med. Microbiol..

[15] Ferry S.A., Holm S.E., Stenlund H., Lundholm R., Monsen T.J. (2007). Clinical and bacteriological outcome of different doses and duration of pivmecillinam compared with placebo therapy of uncomplicated lower urinary tract infection in women: The LUTIW project. Scand. J. Prim. Health Care.

[16] Ejrnaes K., Sandvang D., Lundgren B., Ferry S., Holm S., Monsen T., Lundholm R., Frimodt-moller N. (2006). Pulsed-Field Gel Electrophoresis Typing of *Escherichia coli* Strains from Samples Collected before and after Pivmecillinam or Placebo Treatment of Uncomplicated Community-Acquired Urinary Tract Infection in Women. J. Clin. Microbiol..

[17] Grad Y.H., Lipsitch M., Feldgarden M., Arachchi H.M., Cerqueira G.C., Fitzgerald M., Godfrey P., Haas B.J., Murphy C.I., Russ C. (2012). Genomic epidemiology of the *Escherichia coli* O104:H4 outbreaks in Europe, 2011. Proc. Natl. Acad. Sci. USA.

[18] Zerbino D.R. (2010). Using the Velvet de novo assembler for short-read sequencing technologies. Curr. Protoc. Bioinform..

[19] Treangen T.J., Ondov B.D., Koren S., Phillippy A.M. (2014). The Harvest suite for rapid core-genome alignment and visualization of thousands of intraspecific microbial genomes. Genome Biol..

[20] Seemann T. (2017). Prokka: Rapid prokaryotic genome annotation. Bioinformatics.

[21] Page A.J., Cummins C.A., Hunt M., Wong V.K., Reuter S., Holden M.T.G., Fookes M., Falush D., Keane J.A., Parkhill J. (2015). Roary: Rapid large-scale prokaryote pan genome analysis. Bioinformatics.

[22] Jombart T., Devillard S., Balloux F. (2010). Discriminant analysis of principal components: A new method for the analysis of genetically structured populations. BMC Genet..

[23] Clermont O., Christenson J.K., Denamur E., Gordon D.M. (2013). The Clermont *Escherichia coli* phylo-typing method revisited: Improvement of specificity and detection of new phylo-groups. Environ. Microbiol. Rep..

[24] Wagner A. (2012). Metabolic Networks and Their Evolution. Evolutionary Systems Biology.

[25] Cavicchioli R., Schroder I., Constanti M., Gunsalus R.P. (1995). The NarX and NarQ sensor-transmitter proteins of *Escherichia coli* each require two conserved histidines for nitrate-dependent signal transduction to NarL. J. Bacteriol..

[26] Navarro-Garcia F., Ruiz-Perez F., Cataldi Á., Larzábal M. (2019). Type VI Secretion System in Pathogenic *Escherichia coli*: Structure, Role in Virulence, and Acquisition. Front. Microbiol..

[27] Spurbeck R.R., Stapleton A.E., Johnson J.R., Walk S.T., Hooton T.M., Mobley H.L.T. (2011). Fimbrial profiles predict virulence of uropathogenic *Escherichia coli* strains: Contribution of ygi and yad fimbriae. Infect. Immun..

[28] Lobato-Márquez D., Díaz-Orejas R., García-del Portillo F. (2016). Toxin-antitoxins and bacterial virulence. FEMS Microbiol. Rev..

[29] Lasaro M.A., Salinger N., Zhang J., Wang Y., Zhong Z., Goulian M., Zhu J. (2009). F1C fimbriae play an important role in biofilm formation and intestinal colonization by the *Escherichia coli* commensal strain Nissle 1917. Appl. Environ. Microbiol..

[30] Wright K.J., Seed P.C., Hultgren S.J. (2007). Development of intracellular bacterial communities of uropathogenic *Escherichia coli* depends on type 1 pili. Cell. Microbiol..

[31] Nielsen K.L., Stegger M., Kiil K., Godfrey P.A., Feldgarden M., Lilje B., Andersen P.S., Frimodt-Møller N. (2017). Whole-genome comparison of urinary pathogenic *Escherichia coli* and faecal isolates of UTI patients and healthy controls. Int. J. Med. Microbiol..

[32] Stegger M., Leihof R.F., Baig S., Sieber R.N., Thingholm K.R., Marvig R.L., Frimodt-Møller N., Nielsen K.L. (2020). A snapshot of diversity: Intraclonal variation of *Escherichia coli* clones as commensals and pathogens. Int. J. Med. Microbiol..

