# Supplementary material for: Escherichia coli Causing Recurrent Urinary Tract Infections: Comparison to Non-Recurrent Isolates and Genomic Adaptation in Recurrent Infections
Source: Microorganisms. 2021 Jun 30;9(7):1416. doi: 10.3390/microorganisms9071416 (PMC8303582; doi:10.3390/microorganisms9071416)
Supplement: Supplementary file 1 [file microorganisms-09-01416-s001.zip › microorganisms-1214379-supplementary.pdf]

Supplementary tables

Table S1: Overview of isolates in the study: Infection type, phylogroup, MLST clonal complex (CC), sequencing method, average sequencing depth, number of scaffolds and Genbank/ENA ID

Isolate ID	Infection type	Phylogroup	MLST CC	Sequencing method	Average seq. depth	# of scaffolds	GenBank or ENA ID
3014-1	rUTI	B2	73	PE + MP	142	10	NCBI:txid1281173
3014-3	rUTI	B2	73	PE + MP	273	13	ENA:PRJEB40120
3033-1	rUTI	D	38	PE + MP	138	11	NCBI:txid1281175
3041-1	Cure	B2	95	PE + MP	145	10	NCBI:txid1281176
3052-1	Cure	A	10	PE + MP	246	11	NCBI:txid1281177
3053-1	rUTI	B2	14	PE + MP	137	16	NCBI:txid1281178
3053-3	rUTI	B2	14	PE + MP	278	41	ENA:PRJEB40120
3087-1	Cure	B2	73	PE + MP	138	16	NCBI:txid1281180
3088-1	rUTI	B2	14	PE + MP	141	7	NCBI:txid1281181
3097-1	rUTI	B2	127	PE + MP	256	8	NCBI:txid1281182
3097-3	rUTI	B2	127	PE + MP	121	6	ENA:PRJEB40120
3108-1	Cure	B2	singleton	PE + MP	141	38	NCBI:txid1281183
3113-1	rUTI	B2	127	PE + MP	141	13	NCBI:txid1281184
3113-3	rUTI	B2	127	PE + MP	110	17	ENA:PRJEB40120
3117-1	Cure	B2	420	PE + MP	144	7	NCBI:txid1281185
3121-1	Cure	B2	73	PE + MP	137	13	NCBI:txid1281186
3122-1	Cure	B2	14	PE + MP	231	28	NCBI:txid1281187
3139-1	Cure	A	10	PE + MP	179	21	NCBI:txid1281189
3140-1	Cure	B2	95	PE + MP	135	10	NCBI:txid1281190
3144-1	Cure	D	69	PE + MP	260	3	NCBI:txid1281191
3148-1	Cure	A	10	PE + MP	138	13	NCBI:txid1281192
3150-1	Cure	A	10	PE + MP	131	11	NCBI:txid1281193
3151-1	Cure	A	10	PE + MP	145	8	NCBI:txid1281194
3152-1	Cure	B2	713	PE + MP	138	19	NCBI:txid1281195
3155-1	Cure	B2	1851	PE + MP	142	10	NCBI:txid1281196
3160-1	Cure	B2	372	PE + MP	138	12	NCBI:txid1281198
3161-1	Cure	B2	73	PE + MP	137	16	NCBI:txid1281199
3162-1	rUTI	B2	95	PE + MP	135	16	NCBI:txid1281200
3162-3	rUTI	B2	95	PE + MP	243	14	ENA:PRJEB40120
3163-1	Cure	D	38	PE + MP	144	7	NCBI:txid1281201
3172-1	Cure	B2	73	PE + MP	128	13	NCBI:txid1281202
3173-1	Cure	B2	73	PE + MP	97	16	NCBI:txid1281203
3174-1	rUTI	A	10	PE + MP	138	31	NCBI:txid1281204
3175-1	Cure	B2	73	PE + MP	138	11	NCBI:txid1281205
3176-1	Cure	B1	278	PE + MP	142	11	NCBI:txid1281206
3178-1	rUTI	B2	73	PE + MP	113	19	NCBI:txid1281207

3180-1	Cure	A	10	PE + MP	147	17	NCBI:txid1281208
3185-1	Cure	B2	12	PE + MP	141	9	NCBI:txid1281209
3190-1	rUTI	F	59	PE + MP	116	31	NCBI:txid1281210
3199-1	Cure	A	10	PE + MP	144	6	NCBI:txid1281212
3200-1	Cure	D	31	PE + MP	142	12	NCBI:txid1281213
3203-1	Cure	B2	95	PE + MP	122	20	NCBI:txid1281215
3206-1	Cure	B2	95	PE + MP	145	12	NCBI:txid1281216
3212-1	rUTI	A	693	PE + MP	141	21	NCBI:txid1281218
3212-3	rUTI	A	693	PE	152	32	ENA:PRJEB40120
3215-1	rUTI	B2	14	PE + MP	141	10	NCBI:txid1281219
3215-3	rUTI	B2	14	PE + MP	156	18	ENA:PRJEB40120
3216-1	rUTI	B2	73	PE + MP	249	13	NCBI:txid1281220
3217-1	rUTI	B2	127	PE + MP	145	7	NCBI:txid1281221
3217-3	rUTI	B2	127	PE	48	175	ENA:PRJEB40120
3220-1	rUTI	B2	73	PE + MP	130	16	NCBI:txid1281222
3221-1	rUTI	B2	80	PE + MP	274	11	NCBI:txid1281223
3221-3	rUTI	B2	80	PE	61	132	ENA:PRJEB40120
3222-1	Cure	B2	73	PE + MP	86	24	NCBI:txid1281224
3230-1	rUTI	B2	80	PE + MP	107	13	NCBI:txid1281225
3230-3	rUTI	B2	80	PE + MP	118	47	ENA:PRJEB40120
3233-1	rUTI	B2	73	PE + MP	137	14	NCBI:txid1281226
3233-3	rUTI	B2	73	PE + MP	132	13	ENA:PRJEB40120
3240-1	Cure	A	10	PE + MP	240	8	NCBI:txid1281227
3244-1	rUTI	B2	12	PE + MP	98	15	NCBI:txid1281228
3244-2	rUTI	B2	12	PE	51	76	ENA:PRJEB40120
3257-1	rUTI	B2	73	PE + MP	137	20	NCBI:txid1281230
3257-3	rUTI	B2	73	PE + MP	205	14	ENA:PRJEB40120
3264-1	rUTI	B2	73	PE + MP	130	13	NCBI:txid1281231
3290-1	rUTI	B2	127	PE + MP	140	7	NCBI:txid1281234
3290-2	rUTI	B2	127	PE + MP	175	14	ENA:PRJEB40120
3292-1	Cure	B1	88	PE + MP	273	26	NCBI:txid1281235
3298-1	rUTI	B2	95	PE + MP	278	13	NCBI:txid1281236
3298-3	rUTI	B2	95	PE + MP	176	13	ENA:PRJEB40120
3304-1	Cure	F	59	PE + MP	127	26	NCBI:txid1281237
3317-1	Cure	A	685	PE + MP	137	6	NCBI:txid1281239
3318-1	Cure	A	10	PE + MP	134	14	NCBI:txid1281240
3323-1	Cure	D	405	PE + MP	146	19	NCBI:txid1281241
3336-1	rUTI	B1	88	PE + MP	143	8	NCBI:txid1281243
3336-3	rUTI	B1	88	PE	56	76	ENA:PRJEB40120
3337-1	rUTI	B2	73	PE + MP	143	8	NCBI:txid1281244
3337-3	rUTI	B2	73	PE + MP	64	130	ENA:PRJEB40120

3341-1	rUTI	B2	127	PE + MP	139	12	NCBI:txid1281245
3342-1	rUTI	B2	73	PE + MP	144	13	NCBI:txid1281246
3355-1	rUTI	C	88	PE + MP	144	8	NCBI:txid1281243
3355-3	rUTI	C	88	PE	56	165	ENA:PRJEB40120
3426-1	rUTI	B2	127	PE + MP	140	12	NCBI:txid1281249
3426-2	rUTI	B2	127	PE + MP	90	8	ENA:PRJEB40120
3490-1	Cure	B2	73	PE + MP	141	9	NCBI:txid1281251
3585-1	Cure	B2	127	PE + MP	142	5	NCBI:txid1281252
3617-1	Cure	B2	127	PE + MP	138	22	NCBI:txid1281255
3652-1	Cure	B2	73	PE + MP	143	7	NCBI:txid1281257
3656-1	rUTI	B2	321	PE + MP	144	14	NCBI:txid1281258
3656-3	rUTI	B2	321	PE + MP	93	18	ENA:PRJEB40120
3662-1	rUTI	B2	95	PE + MP	142	5	NCBI:txid1281259
3671-1	Cure	E	543	PE + MP	139	10	NCBI:txid1281260
3682-1	rUTI	A	47	PE + MP	143	2	NCBI:txid1281274
3682-3	rUTI	A	47	PE	95	93	ENA:PRJEB40120
3687-1	rUTI	B2	73	PE + MP	279	13	NCBI:txid1281262
3687-3	rUTI	B2	73	PE	76	108	ENA:PRJEB40120
3693-1	rUTI	B2	12	PE + MP	137	17	NCBI:txid1281263
3693-3	rUTI	B2	12	PE	77	107	ENA:PRJEB40120
3694-1	Cure	B2	73	PE + MP	142	8	NCBI:txid1281264
3702-1	rUTI	B2	95	PE + MP	142	10	NCBI:txid1281265
3702-2	rUTI	B2	95	PE	84	11	ENA:PRJEB40120
3705-1	Cure	B2	73	PE + MP	133	17	NCBI:txid1281267
3707-1	rUTI	B2	73	PE + MP	122	18	NCBI:txid1281268
3707-3	rUTI	B2	73	PE	66	110	ENA:PRJEB40120
3718-1	rUTI	E	219	PE + MP	143	15	NCBI:txid1281269
3718-3	rUTI	E	219	PE	67	166	ENA:PRJEB40120
3805-1	rUTI	B1	763	PE + MP	129	16	NCBI:txid1281270
3805-3	rUTI	B1	763	PE	84	108	ENA:PRJEB40120
3821-1	rUTI	B2	73	PE + MP	140	17	NCBI:txid1281271
3821-2	rUTI	B2	73	PE + MP	35	130	ENA:PRJEB40120
3834-1	rUTI	B2	95	PE + MP	143	15	NCBI:txid1281272
3834-3	rUTI	B2	95	PE	66	109	ENA:PRJEB40120
3889-1	rUTI	A	10	PE + MP	143	9	NCBI:txid1281273
3889-3	rUTI	A	10	PE + MP	172	18	ENA:PRJEB40120
3893-1	rUTI	B2	95	PE + MP	145	9	NCBI:txid1281274
3899-1	rUTI	F	62	PE + MP	128	36	NCBI:txid1281275
3899-3	rUTI	F	62	PE	73	223	ENA:PRJEB40120
3955-1	rUTI	B2	12	PE + MP	129	16	NCBI:txid1281276
3955-2	rUTI	B2	12	PE	60	100	ENA:PRJEB40120

4076-1	rUTI	B2	706	PE + MP	136	11	NCBI:txid1281278
4076-3	rUTI	B2	706	PE + MP	49	33	ENA:PRJEB40120
4207-1	Cure	B2	73	PE + MP	139	13	NCBI:txid1281279

MP: mate-pair. PE: Paired-end

Supplementary Table S2:

Coding SNPs identified between pairs. Represented is isolate ID, nucleotide position, nucleotide change, variant frequency, sequencing depth, amino acid change, CDS position and annotations. Bold represents NSY SNPs in metabolism genes.

Pair ID	Nucleotide position	Change	Variant frequency	Depth	AA Change	CDS Position	Annotation
3014-3	2,314,170	T -> A	93.0%	71	Q -> L	266	**glucose-1-phosphate thymidylyltransferase**
3014-3	2,305,952	A -> C	100.0%	48	Y -> D	781	glycosyl transferase
3014-3	4,636,166	A -> G	99.2%	127		312	p-hydroxybenzoate octaprenyltransferase
3053-1	3,845,398	T -> C	100.0%	152	E -> G	863	DNA-directed RNA polymerase subunit alpha
3053-1	1,249,338	G -> C	100.0%	124	G -> A	3,89	filamentous hemagglutinin
3053-1	4,051,820	C -> A	100.0%	154	P -> T	517	**outer membrane protein slp**
3053-1	1,502,121	A -> G	95.6%	90	Y -> C	368	**protein hnr**
3053-1	4,874,589	A -> G	100.0%	118	D -> G	86	RNA-binding protein Hfq
3097-3	2,270,360	A -> G	100.0%	53	V -> A	7	**1,4-Dihydroxy-2-naphthoyl-CoA synthase**
3097-3	4,254,994	C -> T	95.8%	39		91	elongation factor Tu 2
3097-3	2,985,889	T -> C	100.0%	72		75	hypothetical protein
3097-3	474,620	T -> C	100.0%	60	N -> D	422	inner membrane protein
3097-3	236,449	C -> A	100.0%	98	R -> L	21	**membrane-bound lytic murein transglycosylase D**
3097-3	3,736,694	A -> G	100.0%	110		514	protein YhjK
3113-3	4,164,063	A -> G	100.0%	22	R -> G	349	fimbrial protein F17
3113-3	242,169	C -> T	100.0%	25		408	molecular chaperone
3162-3	1,959,576	G -> T	97.8%	45	P -> T	109	**fumarate hydratase class II**
3162-3	2,379,550	G -> C	98.3%	59		474	hypothetical protein
3212-3	1,169,781	T -> C	100.0%	103		282	30S ribosomal subunit protein S1
3212-3	3,561,380	C -> A	97.2%	176	G -> V	35	**arginine exporter protein**
3212-3	1,349,047	A -> T	94.8%	96	M -> K	587	CsgBAC operon transcriptional regulatory protein
3212-3	958,145	T -> C	98.0%	98		1,764	host specificity protein J
3212-3	2,347,433	A -> G	98.7%	150		1,716	hypothetical protein
3212-3	3,375,656	G -> T	99.4%	171	R -> S	10	inner membrane protein ygbE
3215-3	1,298,885	T -> G	100.0%	81		216	exonuclease VIII
3215-3	1,298,906	G -> A	98.8%	83		195	exonuclease VIII
3215-3	3,131,657	A -> C	100.0%	139		190	RNA polymerase sigma factor
3217-3	2,495,602	T -> C	100.0%	32		563	Fimbrial protein
3221-3	4,766,086	G -> A	100.0%	88	G -> D	596	DNA-directed RNA polymerase subunit beta
3221-3	955,156	C -> T	100.0%	34		1,107	hypothetical protein

3221-3	3,467,324	T -> C	100.0%	99		207	membrane-bound lytic murein transglycosylase C
3221-3	970,430	T -> G	100.0%	66	L -> R	26	**phospho-2-dehydro-3-deoxyheptonate aldolase, Phe-sensitive**
3221-3	4,934,652	G -> A	100.0%	128		561	transcriptional regulator
3221-3	3,585,984	G -> A	95.1%	82	A -> V	191	type II secretion system protein
3230-3	1,304,661	G -> T	100.0%	62	H -> Q	582	**arginine ABC transporter ATP-binding protein**
3230-3	4,344,222	C -> T	100.0%	85	V -> M	973	cell wall structural complex MreBCD, actin-like component MreB
3230-3	4,339,524	A -> C	100.0%	87	V -> G	842	hypothetical protein
3230-3	4,892,380	C -> A	99.1%	108	P -> Q	842	LacI family transcriptional regulator
3230-3	146,116	T -> C	100.0%	61	T -> A	724	**quinoprotein glucose dehydrogenase**
3230-3	4,502,256	G -> A	97.1%	70	R -> H	1,901	transcriptional regulator
3233-3	1,795,494	C -> T	100.0%	57	D -> N	646	**diguanylate phosphodiesterase**
3233-3	1,120,997	C -> G	100.0%	97		354	membrane protein
3233-3	1,508,991	T -> G	100.0%	83	E -> A	881	**nitrate/nitrite sensor protein NarX**
3233-3	3,889,492	T -> G	100.0%	102	K -> Q	280	protein smg
3233-3	1,021,537	G -> A	100.0%	58	A -> V	1,154	**pyruvate dehydrogenase [ubiquinone]**
3233-3	3,194,001	C -> A	98.7%	78		226	RNA polymerase sigma factor RpoS
3233-3	1,386,539	C -> T	98.6%	74	G -> D	1,058	YcfD protein
3244-2	729,617	G -> A	97.7%	44	A -> V	53	adhesin/virulence factor Hek
3244-2	1,413,787	A -> G	100.0%	46	C -> R	568	**glutamate/aspartate transport system permease gltK**
3244-2	731,996	C -> T	98.5%	65	D -> N	1,084	hemolysin activator HlyB
3244-2	4,828,276	A -> C	100.0%	49		741	hypothetical protein
3257-3	1,686,798	T -> C	100.0%	34	S -> G	190	**glutamate/aspartate periplasmic-binding protein**
3298-3	4,620,832	C -> T	100.0%	50		1,302	formate acetyltransferase
3336-3	2,023,783	G -> A	98.1%	54	P -> L	806	flagellin export apparatus, substrate specificity protein FlhB
3336-3	917,406	G -> A	100.0%	61		636	glutathione ABC transporter ATP-binding protein
3336-3	589,061	CA -> TG	100.0%	54	M -> T	869	hypothetical protein
3336-3	2,634,708	T -> C	97.5%	40	M -> V	268	**malic enzyme**
3336-3	266,731	T -> G	100.0%	40		1,92	protein rhsD
3336-3	266,770	C -> T	100.0%	37		1,959	protein rhsD
3336-3	4,676,207	C -> T	98.1%	54	R -> C	1,189	transcriptional regulator
3337-3	4,282,350	G -> A	100.0%	46	P -> L	1,217	chromosomal replication initiation protein DnaA
3337-3	2,510,734	A -> G	100.0%	39	H -> R	692	**D-lactate dehydrogenase**

3337-3	4,337,726	T -> C	98.4%	63	C -> R	358	LacI family transcriptional regulator
3337-3	221,120	A -> G	90.9%	22	N -> D	64	lipoprotein involved with copper homeostasis and adhesion
3337-3	2,119,151	T -> A	100.0%	43	N -> I	581	**response regulator UvrY**
3337-3	2,119,424	C -> T	92.5%	40	S -> N	308	**response regulator UvrY**
3337-3	3,097,094	T -> A	100.0%	54	E -> V	665	RNA polymerase sigma factor RpoS
3337-3	3,060,790	G -> A	100.0%	42	A -> V	974	transcriptional regulator
3426-2	3,465,283	T -> G	98.7%	76	E -> A	17	hypothetical protein
3656-3	1,540,193	G -> A	92.1%	76		2,472	antigen 43
3682-3	3,602,882	G -> A	100.0%	97	G -> D	416	**catabolite activator**
3682-3	763,749	A -> C	99.0%	104	I -> M	3	**glutamate/aspartate ABC transporter permease**
3682-3	416,240	T -> C	100.0%	115		324	hypothetical protein
3682-3	2,991,136	G -> A	100.0%	105		630	lipoprotein NlpD
3682-3	1,908,705	A -> G	100.0%	99	E -> G	443	membrane protein
3687-3	403,116	T -> C	100.0%	87	T -> A	1,192	**ATP synthase subunit alpha**
3687-3	59,790	G -> A	100.0%	69		156	bifunctional glycosyl transferase/transpeptidase MrcB
3687-3	368,453	A -> C	100.0%	66	V -> G	431	chromosomal replication initiator protein DnaA
3687-3	1,779,654	A -> G	95.2%	62		594	hypothetical protein
3687-3	3,707,611	C -> A	100.0%	111	E -> D	528	**protein icc**
3687-3	3,047,669	G -> T	100.0%	41	R -> S	1,186	**response regulator GlrR**
3693-3	3,330,069	A -> G	100.0%	100		601	DNA-directed RNA polymerase subunit sigma
3693-3	128,690	G -> T	98.1%	52	F -> L	24	**tRNA s(4)U8 sulfurtransferase**
3702-2	3,785,016	G -> A	100.0%	61		52	cytoplasmic glycerophosphodiester phosphodiesterase
3702-2	4,674,909	T -> G	100.0%	85	I -> S	338	hypothetical protein
3707-3	4,976,836	A -> G	100.0%	86		915	EF-P lysine aminoacylase GenX
3707-3	2,370,498	G -> A	100.0%	54	G -> E	398	NTE family protein
3707-3	2,370,560	T -> C	100.0%	60		460	NTE family protein
3707-3	4,976,836	A -> G	100.0%	86		915	Elongation Factor P Lys34 lysyltransferase
3707-3	3,181,723	A -> C	98.5%	66	S -> A	340	**GTP pyrophosphokinase**
3707-3	893,140	T -> C	100.0%	71	C -> R	244	hypothetical protein
3707-3	926,572	A -> G	100.0%	67	N -> D	592	**phospho-2-dehydro-3-deoxyheptonate aldolase, Phe-sensitive**
3707-3	1,724,807	T -> G	100.0%	46	N -> T	1,121	**sulfatase YdeN**
3718-3	2,567,333	A -> G	100.0%	53	V -> A	710	**alpha,alpha-trehalose-phosphate synthase [UDP-forming]**

3718-3	2,573,665	T -> A	100.0%	79		311	ferritin
3718-3	4,940,887	A -> C	100.0%	45	I -> M	15	hypothetical protein
3718-3	2,155,740	G -> T	100.0%	69	D -> Y	2,032	**oxidoreductase**
3805-3	2,702,612	C -> A	94.3%	105		720	colanic acid biosynthesis protein
3805-3	637,707	T -> C	98.9%	92	Q -> R	83	hypothetical protein
3805-3	788,604	A -> G	100.0%	59		1,056	hypothetical protein
3805-3	2,675,813	C -> T	100.0%	82	G -> D	245	hypothetical protein
3805-3	264,305	T -> C	100.0%	73		426	sensor protein ZraS
3821-2	4,512,956	A -> C	98.3%	60	D -> E	216	**frataxin**
3821-2	1,213,273	C -> G	100.0%	72		633	hypothetical protein
3834-3	652,304	G -> C	97.6%	85		339	propionate catabolism operon regulatory protein
3834-3	2,264,219	T -> A	96.2%	26	K -> M	14	**Slp family lipoprotein, RpoE-regulated**
3889-3	2,146,045	A -> G	98.9%	90	V -> A	245	**6-phosphogluconate dehydrogenase, decarboxylating**
3889-3	4,500,014	C -> T	100.0%	86	G -> D	1,799	**Fe(3+) dicitrate transporter fecA**
3889-3	747,301	G -> C	100.0%	52	L -> V	2,071	**fused sensory histidine kinase in two-component regulatory system with KdpE: signal sensing protein**
3889-3	4,043,881	G -> T	99.0%	99		1,53	GTP-binding protein
3889-3	829,340	A -> G	100.0%	82	I -> V	727	hypothetical protein
3889-3	4,502,308	A -> G	100.0%	66	I -> T	545	**iron-citrate transporter transmembrane signal transducer**
3889-3	4,286,013	A -> G	98.6%	69	C -> R	1,708	multidrug resistance protein MdtO
3889-3	1,289,304	C -> T	100.0%	59	G -> S	1,402	**nitrate/nitrite sensor protein narX**
3889-3	878,702	A -> G	100.0%	80	D -> G	398	outer membrane protein X
3889-3	3,604,875	A -> G	93.8%	48	N -> S	1,091	protein rhsB
3889-3	1,170,074	T -> C	100.0%	45	H -> R	2,396	**ribonuclease E**
3889-3	2,871,519	A -> C	100.0%	62	C -> G	367	RNA polymerase sigma factor rpoS
3889-3	2,911,597	G -> T	97.1%	69	S -> I	257	**signal transduction histidine-protein kinase BarA**
3889-3	1,071,643	A -> T	100.0%	60	C -> S	1,627	**tyrosine-protein kinase etk**
3889-3	2,426,760	T -> C	100.0%	87		483	Von Willebrand factor domain putative lipoprotein
3899-3	467,696	G -> T	97.6%	42		639	D-galactose transporter
3899-3	1,615,341	T -> G	100.0%	61	M -> R	902	hypothetical protein
3899-3	1,718,438	T -> A	98.1%	53		373	hypothetical protein
3899-3	3,950,512	G -> A	100.0%	45		669	membrane protein

3899-3	4,055,091	G -> A	100.0%	43	P -> L	1,058	multidrug efflux protein AcrB
3899-3	1,785,273	T -> A	100.0%	53	T -> S	28	**nitrate/nitrite sensor protein NarX**
3899-3	432,332	G -> A	100.0%	54	G -> D	353	pap fimbrial major pilin protein
3899-3	4,369,529	A -> T	100.0%	44	Q -> L	1,286	penicillin-binding protein 1A
3899-3	398,572	T -> C	100.0%	56	I -> V	619	polysialic acid transporter kpsD
3899-3	2,807,447	G -> A	97.9%	48	G -> D	500	**pyruvate kinase**
3899-3	2,822,103	A -> G	100.0%	28	H -> R	227	**tRNA (cmo5U34)-methyltransferase**
3955-2	4,632,702	A -> G	98.6%	70		33	50S ribosomal subunit protein L34
3955-2	2,696,114	C -> T	96.3%	54	A -> T	490	**CDP-diacylglycerol-glycerol-3-phosphate 3-phosphatidyltransferase**
3955-2	1,262,026	A -> G	93.3%	45		297	membrane protein
3955-2	2,318,623	T -> A	100.0%	71	N -> I	1,283	**sensor protein phoQ**
4076-3	2,902,065	A -> C	100.0%	75		94	RNA polymerase sigma factor rpoS
